# Treatment-resistant schizophrenia : the relationship between clozapine plasma concentration and clinical outcome

**DOI:** 10.1192/j.eurpsy.2022.2039

**Published:** 2022-09-01

**Authors:** I. Kammoun, R. Jouini, A. Aissa, R. Boukhchina, Y. Zgueb, E. Khelifa, U. Ouali, R. Jomli, F. Nacef, Z. El Hechmi

**Affiliations:** 1 Razi hospital, Psychiatry A Department, Manouba, Tunisia; 2 Razi hospital, Psychiatry F, manouba, Tunisia; 3 Razi hospital, Psychiatry F Department, manouba, Tunisia

**Keywords:** treatment-resistant schizophrenia, clozapine, clozapine plasma concentration

## Abstract

**Introduction:**

Clozapine is highly effective in patients with treatment-resistant schizophrenia but, to ensure optimal clinical response it is important to optimize its use and this depends on adequate pharmacological monitoring.

**Objectives:**

Evaluate the therapeutic response rate according to clozapine plasma concentration.

**Methods:**

It was a cross-sectional, retrospective and analytical study, carried out over a period of six months, in the F and A psychiatry departments of the Razi hospital in Tunis, including patients followed for treatment-resistant schizophrenia and receiving clozapine. We evaluated the response to clozapine using the Brief Psychiatric Rating Scale (BPRS).

**Results:**

The average age was 37.7 ± 9.4. The mean age of introduction of clozapine was 31 years and the mean time to its introduction was 9.3 years. Clozapine was administered as a single drug in 85% of cases. The mean dose of clozapine was 373 mg/day. The mean of clozapine plasma concentration was 386.5 ng/ml with a minimum of 89 ng/ml and a maximum of 913 ng/ml. The clinical response rate to clozapine was 25% with a BPRS good response threshold value of less than 35. Patients with clozapine levels above the conventional cut-off of 350 ng/ml (n=34) had a response rate of 34.6%. A response rate of 37% was observed in the group of patients with a clozapine plasma concentration interval of 200-350 ng/ml. There was no statistically significant difference in therapeutic response (p=0186)

**Conclusions:**

Our study revealed a therapeutic response variation according to plasma clozapine concentration and showed the existence of a non-negligible and effective response rate.

**Disclosure:**

No significant relationships.

